# Elevated O-GlcNAcylation promotes colonic inflammation and tumorigenesis by modulating NF-κB signaling

**DOI:** 10.18632/oncotarget.3725

**Published:** 2015-03-30

**Authors:** Yong Ryoul Yang, Dae Hyun Kim, Young-Kyo Seo, Dohyun Park, Hyun-Jun Jang, Soo Youn Choi, Yong Hwa Lee, Gyun Hui Lee, Kazuki Nakajima, Naoyuki Taniguchi, Jung-Min Kim, Eun-Jeong Choi, Hyo Youl Moon, Il Shin Kim, Jang Hyun Choi, Ho Lee, Sung Ho Ryu, Lucio Cocco, Pann-Ghill Suh

**Affiliations:** ^1^ School of Life Sciences, Ulsan National Institute of Science and Technology, Ulsan, Republic of Korea; ^2^ Division of Molecular and Life Science, Pohang University of Science and Technology, Pohang, Kyungbuk, Republic of Korea; ^3^ Disease Glycomics Team, Systems Glycobiology Research Group, RIKENMax Planck Joint Research Center, Global Research Cluster, RIKEN, Hirosawa, Wako, Saitama, Japan; ^4^ Cancer Experimental Resources Branch, National Cancer Center, Goyang-si, Gyeonggi-do, Republic of Korea; ^5^ Cellular Signaling Laboratory, Department of Biomedical and Neuromotor Sciences, University of Bologna, Bologna, Italy

**Keywords:** O-GlcNAcylation, O-GlcNAcase, colitis, colitis-associated cancer

## Abstract

O-GlcNAcylation is a reversible post-translational modification. O-GlcNAc addition and removal is catalyzed by O-GlcNAc transferase (OGT) and O-GlcNAcase (OGA), respectively. More recent evidence indicates that regulation of O-GlcNAcylation is important for inflammatory diseases and tumorigenesis. In this study, we revealed that O-GlcNAcylation was increased in the colonic tissues of dextran sodium sulfate (DSS)-induced colitis and azoxymethane (AOM)/DSS-induced colitis-associated cancer (CAC) animal models. Moreover, the O-GlcNAcylation level was elevated in human CAC tissues compared with matched normal counterparts. To investigate the functional role of O-GlcNAcylation in colitis, we used OGA heterozygote mice, which have an increased level of O-GlcNAcylation. *OGA*^+/−^ mice have higher susceptibility to DSS-induced colitis than *OGA*^+/+^ mice. *OGA*^+/−^ mice exhibited a higher incidence of colon tumors than *OGA*^+/+^ mice. In molecular studies, elevated O-GlcNAc levels were shown to enhance the activation of NF-κB signaling through increasing the binding of RelA/p65 to its target promoters. We also found that Thr-322 and Thr352 in the p65-O-GlcNAcylation sites are critical for p65 promoter binding. These results suggest that the elevated O-GlcNAcylation level in colonic tissues contributes to the development of colitis and CAC by disrupting regulation of NF-κB-dependent transcriptional activity.

## INTRODUCTION

The addition of O-linked β-N-acetylglucosamine (O-GlcNAc) monosaccharides to Ser and Thr residues of nuclear, mitochondrial and cytoplasmic proteins is termed O-GlcNAcylation. The addition and removal of O-GlcNAc on target proteins is catalyzed by O-GlcNAc transferase (OGT) and O-GlcNAcase (OGA), respectively [1]. The O-GlcNAc cycling enzymes are expressed ubiquitously and are critical for development in vertebrates. Abnormal amounts of O-GlcNAcylation contribute to the etiology of chronic aging diseases, including cardiovascular disease, type 2 diabetes and Alzheimer's disease [2, 3]. O-GlcNAc-modified proteins play diverse roles in transcriptional regulation, the cell cycle, signaling, stress, and differentiation [1]. Because the cellular roles of O-GlcNAc-modified proteins are perturbed during tumorigenesis, aberrant O-GlcNAcylation was suggested to be closely linked to tumorigenesis [4]. OGT was reported to regulate transcriptional machinery by modifying the many transcription factors involved in cancer-relevant processes [4]. Notably, O-GlcNAc levels are significantly elevated in various cancer types, including those of the breast [5], prostate [6], colon [7], lung [8], pancreas [9] and chronic lymphocytic leukemia [10]. *In vivo* xenograft of breast [11], prostate [6] and pancreatic cancers [9] showed a critical role for O-GlcNAcylation in tumorigenesis or metastasis. However, the effect of O-GlcNAcylation in colorectal cancer (CRC) has not been demonstrated *in vivo.*

CRC is one of the most common malignancies, for which inflammation is an established risk factor [12]. Ulcerative colitis (UC), a common form of inflammatory bowel disease (IBD), is associated with an increased risk of CRC [13]. The NF-κB signaling pathway plays a pivotal role in the development and maintenance of intestinal inflammation [14]. Activation of NF-κB signaling was detected in mucosal IBD cells and colorectal carcinoma patients [15, 16]. Previous studies have suggested that NF-κB signaling plays a key role in linking intestinal inflammation with CRC development [17]. A number of genetic mouse models have demonstrated that aberrant NF-κB signaling in mucosal immune cells or intestinal epithelial cells leads to severe intestinal inflammation and tumors [18-20]. Many reports have shown that NF-κB signaling is affected by O-GlcNAcylation. However, no clear pattern in NF-κB activity in the presence of increased O-GlcNAc levels has been reported. O-GlcNAcylation of RelA/p65, a subunit of NF-κB, is critical for activation of NF-κB signaling in mesangial cells and T- and B-lymphocytes [21, 22]. p65 is O-GlcNAcylated on Thr-352, which is required for transcriptional activity under hyperglycemic conditions [23]. Mutation of two p65 O-GlcNAc sites (T322A and T352A) attenuated the growth of pancreatic ductal adenocarcinoma [9]. Furthermore, transforming growth factor-β-activated kinase1-binding protein (TAB1), an upstream signaling molecule of NF-κB, is O-GlcNAcylated and necessary for activation of NF-κB signaling during the innate immune response [24]. In contrast, several reports have suggested that elevated O-GlcNAcylation attenuates NF-κB signaling activation in rat aortic smooth muscle cells and primary cultured cardiomyocytes [25, 26]. Despite the increasing number of reports of a possible link between O-GlcNAcylation and inflammation, the pathogenetic role of O-GlcNAcylation in IBD and CRC, particularly when associated with chronic inflammation, has yet to be investigated. In this study we used *OGA^+/−^* mice with constitutively elevated colonic O-GlcNAc levels as an experimental model to investigate the role of O-GlcNAcylation in intestinal inflammation and colitis-associated cancer. Our results showed that elevated O-GlcNAcylation significantly enhanced colitis and CAC in the colon by modulating p65 DNA-binding activity.

## RESULTS

### Elevation of O-GlcNAcylation in colitis

**Figure 1:**
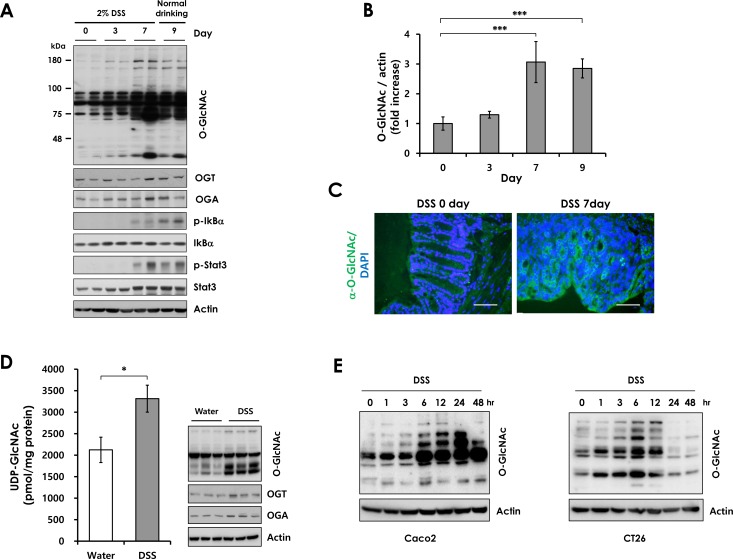
O-GlcNAcylation levels during DSS treatment Wild-type mice were administered 2% DSS in drinking water for the indicated days. (A) Western blots of lysates from wild-type mice treated with DSS during a 9-day time course. Lysates for each time point were isolated from three separate mice. Two representative samples were loaded in each lane. (B) Densitometry was performed on immunoblots. The ratio of O-GlcNAc to β-actin was determined. Error bars represent the S.D. (*n* = 3) and (**) *P* < 0.005, (*) *P* < 0.05 (Student's *t*-test). (C) Immunofluorescence staining of the colon with O-GlcNAc mAb (RL2) in wild-type mice not treated or treated with DSS for 7 days (green, O-GlcNAc modified proteins; blue, nuclei). Bars =50 μm. (D) Measurement of Uridine diphosphate-N-acetylglucosamine (UDP-GlcNAc; B) levels in control (n=3) and DSS-treated (n=6) colon tissues by HPLC analysis (left). Matched O-GlcNAcylation levels (Immunoblots with RL2 antibody) (right). Error bars represent ± SEM. (*) *P* < 0.05 (Student's *t*-test). (E) Caco2 and CT26 cells were treated with 3% DSS for up to 48 hours. O-GlcNAcylation levels were analyzed by Western blot analysis.

Although studies have suggested an association between O-GlcNAcylation and inflammation [24, 25], the involvement of O-GlcNAcylation in IBD has not been investigated. First, we examined whether O-GlcNAcylation is altered in colitis. Wild-type (WT) mice were treated with 2% dextran sulfate sodium (DSS) in drinking water for 7 days to induce colitis, and then provided with normal water. Immunoblotting revealed that global O-GlcNAcylation and O-GlcNAc cycling enzymes were elevated in colonic tissues during DSS-induced colitis (Figure 1A and 1B). OGT expression was consistent with that of OGA during DSS treatment. Apparently, increased OGT expression elevates O-GlcNAcylation, causing an increase in OGA expression to compensate for the increased O-GlcNAcylation. Consistent with previous reports, Iκb and STAT3 were phosphorylated during colitis, indicating development of inflammation [15, 27]. Interestingly, O-GlcNAcylation is increased during colitis, simultaneous with the activation of inflammatory signaling. We also confirmed increased O-GlcNAc levels in colonic tissue of DSS-treated mice using immunohistofluorescence (Figure 1C). To investigate whether UDP-GlcNAc, a donor substrate for O-GlcNACylation, is also elevated in DSS-treated colon tissues, we measured UDP-GlcNAc levels by HPLC analysis in colon tissues after loading control samples from colon tissues (Supplementary Figure 1). UDP-GlcNAc levels are increased in DSS-treated colon tissues compared with control tissues (Figure 1D). This result indicates that hyper-O-GlcNAcylation is caused by both increased flux of the hexosamine pathway and increased OGT expression.

To determine whether DSS affects directly the O-GlcNAc level in colonic epithelial cells, Caco2 and CT26 cells were treated with 3% DSS. DSS treatment increased O-GlcNAcylation in colon epithelial cells (Figure 1E). Notably, high levels of O-GlcNAcase expression was detected in colonic epithelial cells (Supplementary Figure 2). This finding suggests that O-GlcNAc modification might affect DSS-induced colitis progression in intestinal epithelial cells. Furthermore, we also tested whether DSS activates the NF-κB signaling in colonic epithelial cells and MEFs. However, p65 phosphorylation, IκBα phosphorylation, and IκBα degradation were not changed by DSS treatment (Supplementary Figure 3). These results suggest that O-GlcNAc cycling may play an important role in the pathogenesis of colitis.

### Increased levels of O-GlcNAcylation in mouse and human CAC

**Figure 2:**
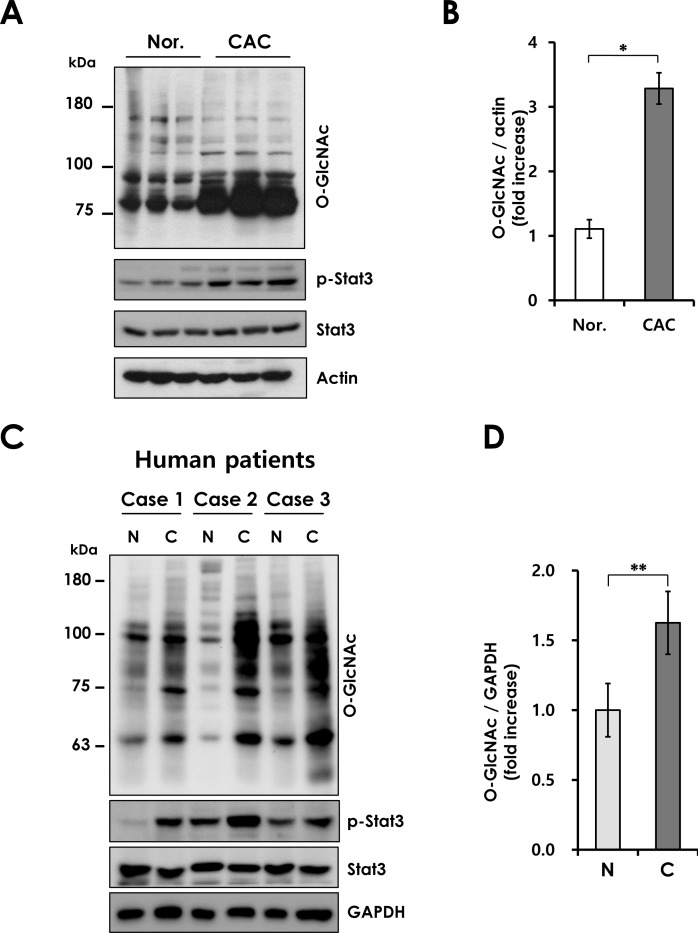
Elevated O-GlcNAcylation in mouse and human CAC (A) Western blot of lysates form wild-type mice not treated or treated with AOM/DSS for 12 weeks. (B) Densitometry was performed on immunoblots. The ratio of O-GlcNAc to β-actin was determined. Error bars represent the S.D. (*n* = 3), (*) *P* < 0.05 (Student's *t*-test). (C) Western blot analysis of O-GlcNAcylation level in 3 matched pairs of uninvolved colonic mucosa (N) and colitis-associated cancer (C) tissues. (D) Densitometry was performed on immunoblots. The ratio of O-GlcNAc to GAPDH was determined. Error bars represent the S.D. (*n* = 3), (**) *P* < 0.005 (Student's *t*-test).

Next, we investigated whether O-GlcNAcylation is also increased in inflammation-associated tumorigenesis. O-GlcNAc levels were assayed in AOM/DSS-induced mouse colon tumors. We observed increased levels of O-GlcNAcylation in AOM/DSS-treated mice. O-GlcNAc levels were elevated in CAC tissues with increased STAT3 phosphorylation, a well-known marker of inflammation, in the AOM/DSS model. (Figure 2A and 2B). These results suggest that O-GlcNAcylation is closely correlated with CAC. To confirm the elevated O-GlcNAcylation in CAC, we used a matched pair of adenomas and uninvolved colonic mucosa from CAC patients. CAC samples show elevated levels of the O-GlcNAcylation and STAT3 phosphorylation compared to uninvolved colonic mucosa (Figure 2C and 2D). The elevated O-GlcNAcylation in CAC tissues might be a consequence of chronic inflammation, as observed in colitis. We speculate that increased O-GlcNAcylation may contribute to the development of CAC.

### OGA^+/−^ mice exhibit increased susceptibility to acute and chronic DSS-induced colitis

**Figure 3:**
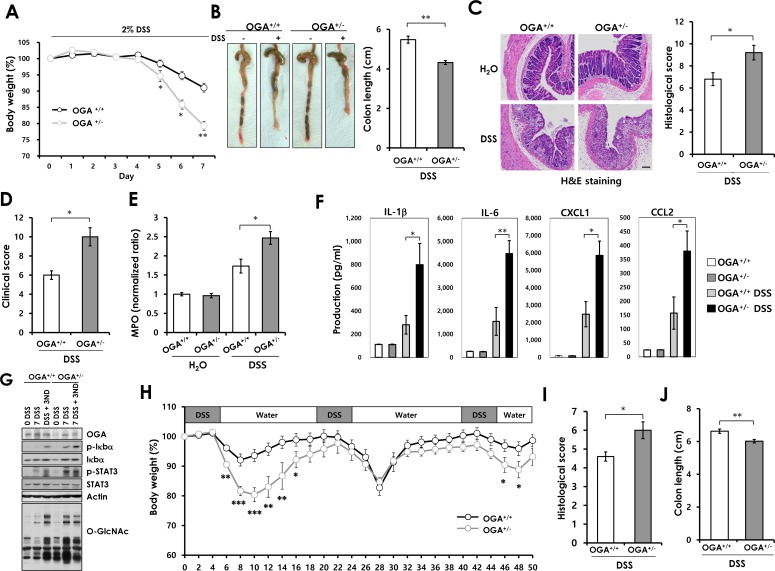
*OGA*^+/−^ mice exhibit increased susceptibility to acute DSS-induced colitis and chronic colitis Eight-week-old *OGA*^+/+^ and *OGA*^+/−^ mice were treated with 2% DSS for 7 days and examined for clinical signs of colitis. (A) Weights of mice during the 7-day treatment period. n = 5 mice per group. (B) Photographs of representative colons and ceca from untreated and DSS-treated *OGA*^+/+^ and *OGA*^+/−^ mice. Colon length, measured at day 7 after treatment. (C) H&E-stained distal colonic sections after 7 days of DSS treatment and histological scores. Bars=100 μm. (D) Clinical scores. (E) MPO activity as a measure of neutrophil infiltration. (F) Cytokines and chemokines measured by Luminex multiplex. n = 5 mice per group. Error bars represent ± SEM. (**) *P* < 0.005, (*) *P* < 0.05 (Student's *t*-test). (G) Colon tissue extracts from *OGA*^+/+^ and *OGA*^+/−^ mice after 7 days of DSS treatment and after 3 days of recovery were immunoblotted with antibodies. (H) The mice were treat with 3 cycles of 3% DSS treatment followed by 14 days of water. The mice were analyzed on day 52. The mean changes in body weight of *OGA*^+/+^ and *OGA*^+/−^ mice. n = 13 mice per group. (**I**) Histological scores (**J**) Colon length. n = 5 mice per group. Error bars represent ± SEM. (**) *P* < 0.005, (*) *P* < 0.05 (Student's *t*-test).

Emerging evidences suggest that O-GlcNAcylation regulates NF-κB signaling which is important for immune hemostasis and inflammation in the intestine. NF-κB signaling controls the expression of factors regulates apoptosis and proliferation during intestinal inflammation [14]. Previous studies show that disruption of NF-κB signaling in epithelium aggravates colitis with severe weight loss, tissue damage, and increased proinflammatory factors in the colonic mucosa [28]. These reports and the above results promoted us to investigate the involvement of O-GlcNAcylation in DSS-induced colitis and colonic inflammation. We used *OGA^+/−^*mice that have constantly elevated O-GlcNAcylation (Supplementary Figure 4A). Homozygous knockout of OGA is lethal perinatally [29]. Colitis was induced in *OGA^+/+^*and *OGA^+/−^*mice by administration of 2% DSS in drinking water for 7 days, followed by normal drinking water. During the DSS treatment period, *OGA^+/−^*mice exhibited more severe body weight loss, with an average weight loss of 8% in *OGA*^+/+^ *versus* 21% in *OGA^+/−^* mice (Figure 3A). The *OGA^+/−^* mice exhibited significantly higher mortality than *OGA*^+/+^ mice during the recovery phase (Supplementary Figure 4B). *OGA^+/−^* mice exhibited more severe colon shortening (Figure 3B). Histological analysis by H&E staining showed that colon tissue from DSS-treated *OGA*^+/+^mice had moderate inflammation and ulceration. However, *OGA^+/−^* mice displayed severe damage with extensive infiltration of neutrophils and a complete loss of crypt architecture (Figure 3C). In addition, *OGA^+/−^* mice showed rectal bleeding than *OGA*^+/+^ littermates, with a higher clinical score (Figure 3D). The extent of neutrophil infiltration was measured by MPO activity assay (Figure 3E). *OGA^+/−^* mice exhibited higher MPO activity than *OGA*^+/+^ mice, indicating increased neutrophil infiltration. Thus, these results suggest that increased O-GlcNAcylation aggravates DSS-induced mucosal damage, as indicated by the greater susceptibility of *OGA^+/−^*mice to DSS treatment. A number of proinflammatory cytokines mediate intestinal inflammation. Therefore, we compared cytokine production in colon samples from *OGA*^+/+^ and *OGA^+/−^*mice before and after DSS treatment. As shown in Figure 3F, *OGA^+/−^*mice exhibited a marked increase in DSS-induced colonic production of proinflammatory cytokines (IL-1β and IL-6) and chemokines (CXCL1 and CCL2). Consistent with these results, activation of the inflammatory pathway including NF-κB and STAT3 signaling, in the colon of DSS-treated *OGA^+/−^* mice was greater than in *OGA*^+/+^ mice due to the elevated O-GlcNAc levels of *OGA^+/−^*mice (Figure 3G).

We next investigated the roles of O-GlcNAcylation in the DSS-induced chronic colitis. The chronic colitis was induced 3 cycles of 3% DSS treatment for 5 days by 14 days of water. Consistent with the results of acute colitis, *OGA^+/−^*mice showed increased susceptibility to chronic colitis compared with *OGA*^+/+^ mice. *OGA^+/−^*mice exhibited increased body weight loss (Figure 3H) and decreased survival rate during chronic colitis progression (Supplementary Figure 5A). Severe colitis of *OGA^+/−^*mice were reflected by histopathological image analysis (Supplementary Figure 5B) with higher histological scores (Figure 3I) and colonic shortening compared to *OGA*^+/+^ mice (Figure 3J). Overall, these results indicate that increased O-GlcNAcylation aggravates intestinal inflammation in a mouse model of colitis.

### Increased O-GlcNAcylation contributes to colitis-associated colorectal cancer

**Figure 4:**
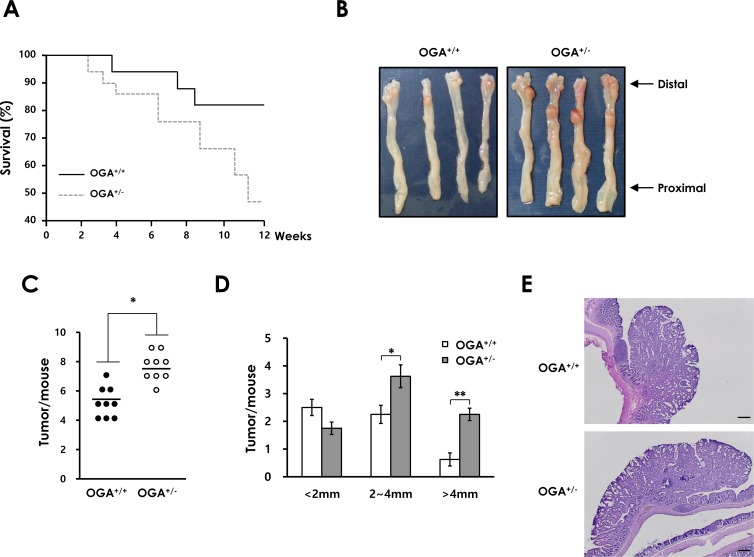
Elevated O-GlcNAcylation contributes to colonic tumorigenesis in a mouse model of CAC (A) *OGA*^+/+^ and *OGA*^+/−^ mice littermate survival (18 of 21 *OGA*^+/+^ mice 9 of 20 *OGA*^+/−^ mice). (B) Representative colons at the end of the CAC protocol. (C) Number of tumors per mouse and (D) tumor size. Size distribution of tumors is shown. (E) Representative H&E-stained colonic tumors at the end of the CAC protocol. Bars =200 μm.

To further characterize the effects of elevated O-GlcNAcylation on DSS-induced colitis, we examined whether increased O-GlcNAcylation promotes the development of CAC. *OGA*^+/+^ and *OGA^+/−^* mice were assessed using the AOM/DSS model of CAC. As expected, *OGA^+/−^* mice exhibited a higher mortality rate than *OGA*^+/+^ mice (Figure 4A). The early death of *OGA^+/−^* mice might be associated with greater susceptibility to DSS-induced colitis as observed in Figure 3 and Supplementary Figure 4. Both *OGA*^+/+^ and *OGA^+/−^* mice developed tumors in the middle-to-distal portion of the colon (Figure 4B). *OGA^+/−^* mice developed a markedly greater number of tumors (Figure 4C). The average tumor size was also significantly greater in *OGA^+/−^* mice than *OGA*^+/+^ mice (Figure 4D). Histological examination consistently showed larger adenomas in colonic sections of *OGA^+/−^* mice compared with *OGA*^+/+^ mice (Figure 4E). These results reflect the result showing reduced survival rate of *OGA*^+/+^ mice. These results indicate that increased O-GlcNAcylation may accelerate tumor development.

### Increased O-GlcNAcylation enhances LPS-induced activation of NF-κB signaling

Intestinal inflammation is caused by a dysregulated mucosal immune response to luminal bacteria. LPS and other bacterial products are recognized Toll-like receptors (TLRs), which lead to NF-κB signaling activation. TLR signaling is an important for luminal bacteria-induced intestinal inflammation [30, 31]. NF-κB signaling activation has been proposed as a link between inflammation and carcinogenesis by acting both in tumor and inflammatory cells to promote tumor development [32]. Notably, the key regulators of NF-κB signaling p65, IKKβ and TAK1, are O-glycosylated and regulated by this modification [23, 24, 33, 34].

**Figure 5:**
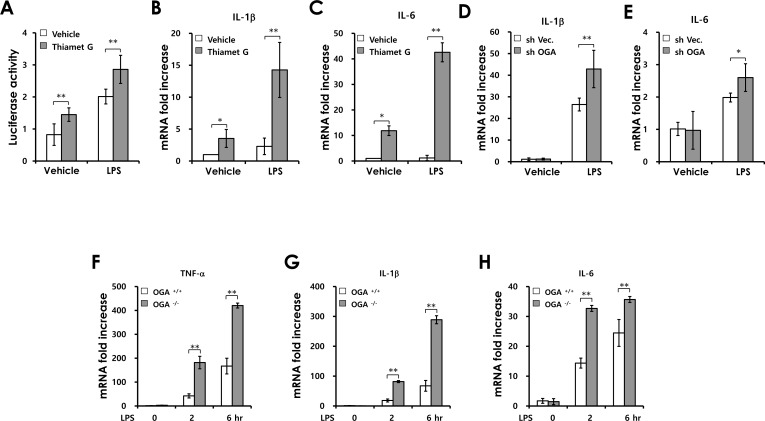
Increased O-GlcNAcylation enhances LPS induced-NF-κB signaling activation (A) CT26 cells were transfected with an NF-κB promoter–reporter construct before LPS (100 ng/mL) and/or Thiamet G (1μM) treatment; luciferase activity was measured at 6 h. (B and C) SYBR Green-based RT-PCR analysis was carried out after treatment with LPS (100 ng/mL) and/or Thiamet G (1 μM) and expression levels of IL-1β and IL-6 were determined by comparison with GAPDH. (D and E) Control and OGA knockdown Caco2 cell lines were stimulated with LPS and the mRNA levels of IL-1β and IL-6 were measured at 6h. (F-H) *OGA*^+/+^ and *OGA*^−/−^ MEFs were stimulated by LPS (100 ng/ml) and the mRNA levels of TNF-α, IL-1β and IL-6 were measured at the indicated times post-stimulation. Error bars represent the S.D. (*n* = 3). (**) *P* < 0.005, (*) *P* < 0.05 (Student's *t*-test.

To investigate how O-GlcNAcylation affects NF-κB signaling in colonic epithelial cells, we examined the effect of increased O-GlcNAcylation on LPS-induced NF-κB activation. We used Thiamet G, a potent and selective inhibitor of OGA (Supplementary Figure 6). As shown in Figure 5A, Thiamet G enhanced LPS-induced NF-κB reporter gene activation in CT26 colonic epithelial cells. Consistent with this result, the expression of NF-κB target genes (IL-1β and IL-6) induced by LPS stimulation was increased in Thiamet G-treated cells (Figure 5B and 5C). We also observed that OGA knockdown promotes LPS-induced IL-1β and IL-6 mRNA production compared with control cells (Figure 5D and 5E). To further confirm the effect of increased O-GlcNAcylation, we used *OGA*^−/−^ mouse embryonic fibroblasts (MEFs). Similarly, the expression of NF-κB target genes was significantly increased in *OGA*^−/−^ MEFs after LPS treatment compared with in wild-type (WT) MEFs (Figure 5F-5H). These results indicate that increased O-GlcNAcylation promotes LPS-induced activation of NF-κB signaling in colonic epithelial cells and MEFs. These data suggest that dysregulation of O-GlcNAcylation contributes to the development of colitis and CAC through enhancing activation of inflammatory pathway in intestinal epithelial cells.

### NF-κB p65 subunit binding to promoter regions is regulated by O-GlcNAcylation

**Figure 6:**
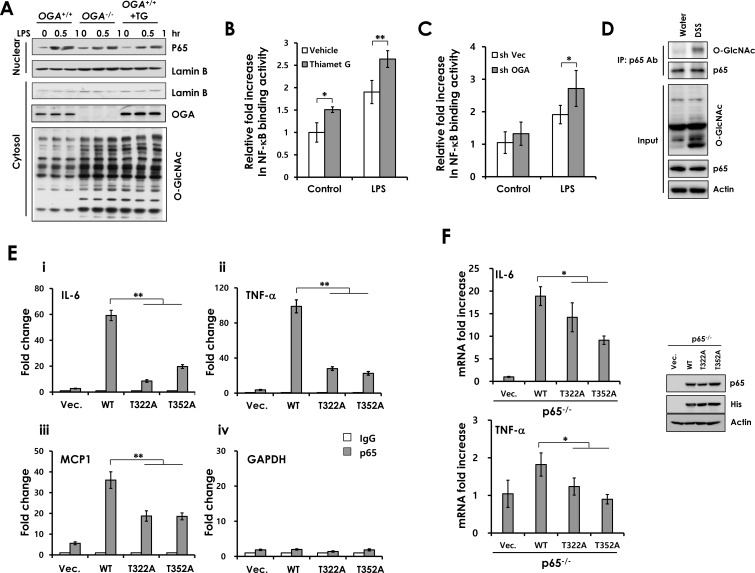
The O-GlcNAcylation site of p65 is important for binding of p65 to the promoter elements (A) *OGA*^+/+^, *OGA*^−/−^, and Thiamet G-treated- *OGA*^+/+^ MEFs were stimulated with LPS. The nuclear extracts were prepared and subjected to Western blot analysis. (B) NF-κB p65 subunit DNA binding activity was analyzed with nuclear extracts isolated from control and Thiamet G pre-treated Caco2 or (C) control and OGA knockdown Caco2 cell lines. (D) Water-treated or DSS-treated colon homogenates were used for immunoprecipitation with a polyclonal p65 antibody and immunoblotted against O-GlcNAc. The levels of p65 expression and O-GlcNAcylation were detected in input homogenates. Recruitment of p65 on the (E) (i) IL-6, (ii) TNF-α, (iii) MCP1 promoter, and (iv) GAPDH (control) were measured by p65 ChIP assay in HEK-293 cells expressing p65 WT, p65T322A or p65T352A. (F) p65 KO MEFs were transfected with plasmids containing p65 WT, p65T322A, p65T352A, or empty vector. RT-PCRs were performed to detect the p65 target genes, IL-6 and TNF-α. The expression of these plasmids expressing WT or mutant p65 were determined by immunoblotting with anti p65, His, and β-actin antibodies (right). Error bars represent the S.D. (*n* = 3). (**) *P* < 0.005, (Student's *t*-test).

In subsequent experiments, we investigated the effect of O-GlcNAcylation on the NF-κB signaling pathway by western blot. LPS treatment rapidly induced Iκb phosphorylation, Iκb degradation and p65 phosphorylation in both control and OGA knockdown Caco2 cells (Supplementary Figure 7A). We observed the same results in *OGA*^+/+^ MEFs and *OGA*^−/−^ MEFs (Supplementary Figure 7B). Since O-GlcNAcylation did not affect Iκb degradation and p65 phosphorylation, we next focused on p65 nuclear translocation. We found that elevated O-GlcNAcylation did not affect p65 nuclear translocation in OGA knockdown and Thiamet G-treated Caco2 cells (Supplementary Figure 7C) and found an identical result in *OGA*^−/−^ MEFs and Thiamet G-treated *OGA*^+/+^ MEFs (Figure 6A).

To determine whether elevated O-GlcNAcylation enhances DNA binding of p65, we performed a p65-binding activity assay using nuclear extracts isolated from control Caco2 cells and Thiamet G-treated Caco2 cells. LPS significantly increased the binding activity of p65 in the control extract. LPS-induced p65 binding activity was significantly enhanced in extracts both Thiamet G-treated cells and OGA knockdown Caco2 cells (Figure 6B and 6C). These results indicate that O-GlcNAcylation regulates p65 DNA-binding activity. Accordingly, we tested whether p65 was highly O-GlcNAcylated in DSS-treated colonic extracts. We detected elevated p65 O-GlcNAcylation in DSS-treated colon compared with control (Figure 6D).

Next, we investigated whether p65 O-GlcNAcylation affects promoter recruitment of p65 using chromatin immunoprecipitation (ChIP) assay. A previous study reported that Thr-322 and Thr-352 of p65 are O-GlcNAcylated [23]. We determined whether Thr-322 or Thr-352 mutation disrupts p65 binding to target promoters. HEK293 cell were transfected with WT p65 or His-p65 mutants (T322A and T352A). The O-GlcNAc level of p65 mutants was reduced slightly in the presence of identical amounts of each expression plasmid (Supplementary Figure 8). WT p65 was recruited to IL-6, TNF-α and MCP-1 promoter elements. However, mutation of Thr-322 or 352 to alanine reduced p65 binding to target promoters (IL-6, TNF-α and MCP1; Figure 6E). Consistent with these data, overexpression of p65 O-GlcNAcylation mutants in p65 KO MEFs exhibited reduced mRNA expression of IL-6 and TNF-α (Figure 6F). These findings indicate that p65 O-GlcNAcylation on Thr-322 and Thr-352 is required for transcriptional activation of p65 and suggest that elevated p65 O-GlcNAcylation promotes NF-κB signaling by modulating promoter recruitment of p65.

## DISCUSSION

Many studies have suggested that NF-κB signaling is a critical link between inflammation and carcinogenesis. Although the involvement of O-GlcNAcylation in NF-κB signaling has been suggested, the molecular mechanism and physiological roles have not been elucidated. In this study, we showed that O-GlcNAcylation is elevated in intestinal inflammation. Notably, O-GlcNAcylation levels were elevated in mouse and human CAC, suggesting that O-GlcNAcylation contributes to chronic inflammation and tumor development. We showed that increased O-GlcNAcylation enhances sensitivity to DSS-induced colitis and promotes CAC by modulating NF-κB signaling. O-GlcNAcylation of p65 affects its binding to target promoters. These results suggest a role for O-GlcNAc cycling in the pathogenesis of inflammatory bowel diseases and colorectal cancer.

Previous studies showed that NF-κB signaling is regulated by O-GlcNAcylation differently. O-GlcNAcylation of the Iκ-B kinase subunit β at Ser733, an inactivating phosphorylation site, is required for catalytic activity in response to high-glucose conditions [33]. The NF-κB p65 subunit is O-GlcNAcylated at Thr-305, Thr-322 and Thr-352. Point mutation of these sites revealed that O-GlcNAc modification at Thr-352 regulates p65 transcriptional activity [23]. In addition, Thr-305 is essential for p65 transcriptional activity by potentiating p300-mediated acetylation [35]. These studies demonstrated that O-GlcNAcylation of both IKK and NF-κB p65 enhances the transcriptional activity of NF-κB signaling under hyperglycemic conditions. In contrast, other studies have reported that elevated O-GlcNAcylation suppressed NF-κB signaling. Glucosamine (GlcN), a major product of the hexosamine biosynthesis pathway, or PUGNAC (OGA inhibitor) treatment inhibits TNF-α–induced NF-κB signaling in vascular smooth-muscle cells by blocking NF-κB p65 Ser 536 phosphorylation [25]. In rat trauma hemorrhage, increased O-GlcNAc modification in the heart, liver and kidney improved cardiac function and organ perfusion with reduced activation of NF-κB signaling [26, 36]. Although reported in many studies, the role of O-GlcNAcylation in NF-κB signaling is dependent on the experimental design and conditions used. In the present study, we showed that increased O-GlcNAcylation caused by OGA deletion or knockdown did not affect Iκb degradation or p65 phosphorylation and nuclear translocation (Supplementary Figure 7). However, elevated O-GlcNAcylation affected NF-κB p65 transcriptional activity by modulating p65 DNA binding (Figure 6B). Regarding phosphorylation of Iκb, this *in vitro* result is inconsistent with the *in vivo* data shown in Figure 3G. We speculate that elevated p65 promoter-binding activity eventually aggravate colonic inflammation in DSS-treated OGA^+/−^ mice. Consequently, OGA^+/−^ mice exhibited increased Iκb and STAT3 phosphorylation compared with WT mice during colitis. Our finding is supported by reports of involvement of many O-GlcNAc–modified proteins in transcriptional regulation: c-myc [37], FoxO-1 [38], PGC-1α [39], and HCF-1 [40].

Recent studies have suggested that dysregulation of O-GlcNAcylation is associated with tumor growth and metastasis. Correlatively, aberrant O-GlcNAcylation and protein levels of O-GlcNAc cycling enzymes are observed in tumors. O-GlcNAcylation levels are elevated in breast, lung, chronic lymphocytic leukemia, and colon tumors [5, 8, 10]. Consistent with these reports, we found that O-GlcNAcylation was elevated in the colon tissues of CAC mice, and OGA^+/−^ mice showed an increased incidence of colorectal tumors compared to control mice. Evidently, NF-κB signaling contributes to cancer development and progression. Colon cancer cell lines and human tumor samples show increased activity of NF-κB, which is a crucial mediator of inflammation-induced tumor growth and progression [41, 42]. Based on these results, we suggest that elevation of O-GlcNAcylation aggravates chronic inflammation by enhancing NF-κB signaling activation, which contributes to CAC development. In contrast, OGA^+/−^ mice were protected from tumor development in Apcm^in/+^ model of sporadic CRC [43]. This finding suggests that increased O-GlcNAcylation suppresses colorectal tumorigenesis. This observation indicates that O-GlcNAcylation differentially affects sporadic and inflammatory-driven tumorigenesis. We suggest that colitis-associated cancer which is different state of inflammation compared with sporadic cancer requires hyper-O-GlcNAyclation for enhancing NF-κB signaling in tumor growth and progression. Previous studies also suggest that the opposite effects on colorectal tumorigenesis in different types of colorectal cancer models is responsible for involvement of different signaling pathway [44, 45].

In addition to O-GlcNAcylation modulating NF-κB signaling, the effects of the many transcriptional mediators that are O-GlcNAcylated in colorectal tumorigenesis cannot be disregarded. Dysregulation of O-GlcNAcylation alters the activity of transcriptional mediators that contribute to tumor growth and metastasis [4, 46]. Consistent with these reports, reduced O-GlcNAcylation of FoxM1, an oncogenic transcription factor, *via* OGT knockdown in breast cancer cells leads to inhibition of tumor growth both *in vitro* and *in vivo* [11]. Furthermore, phosphofructokinase 1 (PFK1) is O-GlcNAcylated. Inhibition of PFK1 O-GlcNAcylation blocks tumor growth *in vivo* [47]. Additionally, a proteomic study newly identified aberrant O-GlcNAcylated proteins involved in the stress response, biosynthesis, RNA metabolism, gene expression, cytoskeleton, and primary breast malignant tumors [48].

In summary, we demonstrated the role of O-GlcNAcylation in colitis and CAC using OGA^+/−^ mice. Our results indicate that O-GlcNAc cycling is critical for regulation of NF-κB signaling in colitis and CAC. Therefore, we propose that dysregulation of O-GlcNAcylation is involved in diverse inflammatory diseases, and thus pharmacological modulation of O-GlcNAc cycling enzymes could be a novel therapeutic strategy for such conditions.

## MATERIALS AND METHODS

### Mice

*OGA^+/−^* mice (C57BL/SV129) were generated as described previously [29]. *OGA^+/−^* mice were backcrossed with C57BL/6J wild-type mice. Mouse strains were bred and housed in the Animal Research Facility at POSTECH and UNIST.

### Human patient samples

The biospecimens for this study were provided by the Pusan National University Hospital, a member of the National Biobank of Korea, which is supported by the Ministry of Health and Welfare. All samples derived from the National Biobank of Korea were obtained with informed consent under institutional review board-approved protocols. All patients were included based on clinical, endoscopical, and pathological proven by gastroenterologists. Four tissue specimens were obtained from patients with CAC. A part of the colon with the cancer and a small segment of normal colon were obtained from patients with CAC.

### Colitis and CAC induction

To induce colitis, 7–8-week-old sex-matched *OGA*^+/+^ and *OGA^+/−^* mice were provided with 2% DSS (molecular weight, 35,000–50,000; MP Biomedicals, Solon, OH) in drinking water *ad libitum* for 7 days. CAC was induced as described previously [49], with some modifications since C57Bl/6J mice show low susceptibility to AOM/DSS-induced CAC [50]. Mice were intraperitoneally injected with AOM (10 mg/kg body weight) and maintained on a regular diet and water for 7 days (*OGA*^+/+^ and *OGA^+/−^* mice). Mice were then subjected to five cycles of DSS treatment, in which each cycle consisted of 1.5% DSS for 7 days (*OGA*^+/+^ and *OGA^+/−^* mice) followed by a 7-day recovery period with regular water.

### Clinical activity score

Body weight loss, stool consistency and the presence of occult/gross blood were assessed daily for each mouse as described previously [51], with some modifications. Body weight change was scored as follows: 0, no change; 1, 1–5% weight loss; 2, 5–10% weight loss; 3, 10–20% weight loss; 4, >20% weight loss. Stool character was scored as follows: 0, normal; 1, soft with well-formed pellets; 2, soft without pellets; 3, diarrhea. Occult blood was scored as follows: 0, no blood; 2, blood; 3, gross bleeding. These scores were added to generate a clinical activity score ranging from 0 to 10.

### Myeloperoxidase (MPO) activity

MPO activity was determined using the EnzChek MPO Activity Assay Kit (Invitrogen) according to the manufacturer's instructions. Briefly, the colon was homogenized in MPO assay buffer and the homogenate was centrifuged at 13000× *g* for 15 min at 4°C. Supernatants were used for assay. The protein concentrations in all tissue extracts were adjusted to 1 mg/ml. The absorbance at 412 nm was measured using a spectrophotometer.

### Cytokine measurements

To assay cytokine levels in colon tissue, a part of the colon was homogenized mechanically in PBS containing 0.1% Triton-X100 and a complete protease inhibitor mixture. Mouse cytokines and chemokines in colon homogenates were determined by Luminex assay (Bio-Rad, Hercules, CA).

### Measurement of UDP-GlcNAc

UDP-GlcNAc levels were determined using by HPLC analysis of extracts of colon tissue samples as previously described [52]. Briefly, colon tissues are homogenized in 70% ice-cold ethanol. Then the lysates are further lysed on the ice using a handy sonic disruptor. The extract was centrifuged at 16,000 × g for 10 min at 4°C in order to remove insoluble material, and the supernatant was lyophilized. Purification of the extracted nucleotide sugars, and separation and quantification by ion pair reversed phase HPLC, have already been reported in detail previously [52].

### Cell culture and generation of MEFs

The human (Caco-2) and mouse (CT26) colonic epithelial cell lines, and RAW264.7 cell lines were purchased from the American Type Culture Collection (ATCC); (Caco2, 2010), (CT26, 2010), and Cell lines were cultured in the recommended ATCC media. Cell line authentication was done by ATCC. Cells were daily checked by morphology and tested to be Mycoplasma free by PCR 1 weeks after recovery from frozen stocks in culture. These cell lines were routinely cultured at 37°C in 5% CO_2_. The cultures were maintained for no longer than 8 weeks after recovery from frozen stocks in culture. Primary MEFs were isolated from 12.5–14.5 dpc embryos from heterozygous mice breeding. After removal of the intestinal organs and head, embryos were washed with phosphate-buffered saline (PBS), minced and trypsinized. After centrifugation, the dissociated cells were plated in medium. The medium was composed of DMEM glutamax, 10% FBS, Pen/Strep and 1% non-essential amino acids. Genotyping was done by both PCR and Western blot. Early passage (P<4) MEFs were used for all experiments.

### Western blotting

Tissue and cell lysates were prepared using standard procedures. Samples of 20–30 μg of protein were separated on an SDS-polyacrylamide gel and visualized. Western blotting antibodies used were anti-phospho-IκB-α (9246; Cell Signaling Technology), anti-IκB-α (4812; Transduction Laboratories), anti-Stat3 (Transduction Laboratories), anti-phospho-Stat3 (9131; Cell Signaling Technology), anti-phoshpo-p65 (3036; Cell Signaling Technology), anti-p65 (ab7970; Abcam), anti-Lamin B (6216; Santa Cruz Biotechnology Inc.), anti-O-GlcNAc (CTD110.6)(9875; Cell Signaling Technology), anti-O-GlcNAc (RL2)(MA1-072; Thermo), and β-actin (691001; MP Biomedicals). Anti-OGA polyclonal antibodies had been previously generated and were used as previously described.[53] The peroxidase-labeled goat anti-rabbit IgG and the goat anti-mouse IgA, IgM, and IgG used as the secondary antibodies were obtained from Kirkegaard & Perry Laboratories (KPL, Gaithersburg).

### NF-κB transcriptional activity assay

To assay NF-κB transcriptional activity, colonic epithelial cells were seeded (1×10^4^/well) in a 24-well plate. After reaching 60% confluence, the cells were transiently transfected with 0.5 μg of NF-κB-luciferase promoter-reporter construct (pGL4.32 [luc2P/NF-κB -RE/Hygro]) and 0.1 μg of control reporter plasmid (pRL-TK), containing the *Renilla reniformis* luciferase gene downstream of the TK promoter. Transfections were carried out using Lipofectamine (Invitrogen) as a transfection reagent. Twenty-four hours after transfection, the cells were treated with LPS, washed with ice-cold PBS and harvested in reporter lysis buffer. Luciferase activity was measured using the Dual Luciferase Assay System. All experiments were performed in triplicate and relative luciferase activity was reported as the fold induction after normalization for transfection efficiency.

### Statistical analysis

Data are presented as means ± standard errors of the means (SEMs) or standard deviation (SD) as indicated in the figure legends. Comparisons between two groups were made by unpaired two-tailed Student's t tests. P values of <0.05 were considered statistically significant. Microsoft Excel was used for statistical calculations.

Histological assessment of colitis, NF-κB binding activity assay, ChIP assay, LPS injection and mouse model of endotoxin shock and shRNA knockdown and stable cell line generation.

See Supplementary Materials and Methods section.

## SUPPLEMENTARY MATERIAL FIGURES AND TABLES


