# Supplementary material for: Elevated O-GlcNAcylation promotes colonic inflammation and tumorigenesis by modulating NF-κB signaling
Source: Oncotarget. 2015 Mar 30;6(14):12529–42. doi: 10.18632/oncotarget.3725 (PMC4494956; doi:10.18632/oncotarget.3725)
Supplement: Supplementary file 1 [file oncotarget-06-12529-s001.pdf]

Elevated O-GlcNAcylation promotes colonic inflammation and tumorigenesis by modulating NF-κB signaling

SUPPLEMENTAL MATERIALS AND METHODS

Histological assessment of colitis

H&E-stained colonic sections were coded for blind microscopic assessment of inflammation (i.e., DSS-induced colitis). Histological scoring was based on 3 parameters. Severity of inflammation was scored as follows: 0, rare inflammatory cells in the lamina propria; 1, increased numbers of granulocytes in the lamina propria; 2, confluence of inflammatory cells extending into the submucosa; 3, transmural extension of the inflammatory infiltrate. Crypt damage was scored as follows: 0, intact crypts; 1, loss of the basal one-third; 2, loss of the basal two-thirds; 3, entire crypt loss; 4, change of epithelial surface with erosion; 5, confluent erosion. Ulceration was scored as follows: 0, absence of ulcer; 1, 1 or 2 foci of ulcerations; 2, 3 or 4 foci of ulcerations; 3, confluent or extensive ulceration. Values were added to give a maximal histological score of 11.

NF-κb binding activity assay

p65 binding activity was measured by using the TransAM™ NF-κB p65 assay kit (Active Motif) according to the manufacturer's instructions. Briefly, the assay measures the nuclear binding of p65 to a consensus NF-κB binding site. Nuclear extracts were isolated by using the NE-PER Nuclear Protein Extraction Kit (Thermo). Ten micrograms of nuclear extracts were used for the assay.

ChIP assay

ChIP procedures for p65 were performed by a combination of published protocols(1,2). Shortly, HEK 293T cells(hereafter 293T cell) were seeded onto 150 mm-culture dishes in DMEM containing 10% FBS and were transfected with pCDNA3.1- 6x his, pCDNA3.1- 6x his -p65, pCDNA3.1- 6x his -p65(T322A), and pCDNA3.1- 6x his -p65(T352A), respectively. p65 WT and mutant expressing plasmid was kindly provided by Dr. Jin Won Cho (Yonsei University, Republic of Korea).

Next day, cells were collected by scrapping and were lysed (2% SDS, 5% β-mercaptoethanol, 125 mM Tris–HCl, pH 6.8, 20% glycerol) and followed for ChIP procedure . 293T cells were crosslinked for 10 min by adding formaldehyde to a final concentration of 1% with mild agitation. Crosslinking was stopped by the addition of glycine to a final concentration of 125 mM, and cells were washed three times with ice-cold PBS prior to harvesting by scraping of the plates. Chromatid were fragmented for 20 min (10 sec on pulse and 20 sec off pulse) to produce fragments ~500 nt in size using the Bioruptor sonicator (Diagenode). Antibody (ab9108; Abcam) to His was used to pull down target chromatid from1 × 108 cells. Immunoprecipitated cross-linked complexes were incubated with protein A-agarose (Upstate Biotechnology), washed sequentially with low salt buffer, high salt buffer and LiCl buffer and eluted from the protein A-agarose bead. After reversing the cross-linking, DNA was isolated from proteinase K-digested samples and PCR was performed to amplify the target promoter region. ChIP chromatid were tested by qPCR using positive and negative control primer sets shown in Table 1.

Table 1. Primers used for ChIP assay

Target Genes	Foward primers	Reverse Primers
GAPDH	TACTAGCGGTTTTACGGGCG	TCGAACAGGAGGAGCAGAGAGCGA
IL6	CGGTGAAGAATGGATGACCT	AAACCAGACCCTTGCACAAC
MIP-2	CGGGAGTTACGCAAGACAGT	ACCCCTTTTATGCATGGTTG
MCP-1	CCCATTTGCTCATTTGGTCT	CTTATTGAAAGCGGGCAGAG
TNF a	GTCTCCGGGTCAGAATGAAA	AGTCCTGAGGCCTGTGTTTG
OGT	ATGGATTTTGACGAACGTG	GTTGGCCGTATAGCCTGAAA

Quantitative real-time PCR for ChIP and mRNA expression; 3 ul out of 200ul of purified genomic chrpmatid pull downed with flag antibody was used as a template for quantitative real-time PCR with conditions: 95°C for 2 min followed by 40 cycles at 95°C for 15 s and 60°C for 31 s. The PCR was carried in LightCycler 480II(ROCHE) using the premixed 2× real-time SYBR Green reagent (Roche).

shRNA knockdown and stable cell line generation

Lentiviral nontarget shRNA plasmids (SHC002: CCGGCAACAAGATGAAGAGCA CCAACTCGAGTTGGTGCTCTTCATCTTGTTGTTTTT), shRNA lentiviral plasmids targeting human OGA (TRCN0000134040: CCGGCCAGAAACTTTCCTTGCTAATCTCG AGATTAGCAAGGAAAGTTTCTGGTTTTTTG). Caco2 cells in 10-cm^2 dishes were transfected with lipofectamine (Invitrogen, Carlsbad, CA, USA) according to the manufacturer's instructions. Cells were cultured in puromycin-containing medium (2 μg/mL) for 5 days to select stable cell lines. To minimize batch-to-batch differences, we compared only cells made from the same batch of cells.

LPS injection and mouse model of endotoxin shock

LPS (Sigma-Aldrich) derived from *Escherichia coli* 0111:B4 were diluted in sterile PBS. Mice (8–10 weeks old) were injected intraperitoneally with 20 mg/kg LPS. Mortality was monitored for 4 days after LPS injection.

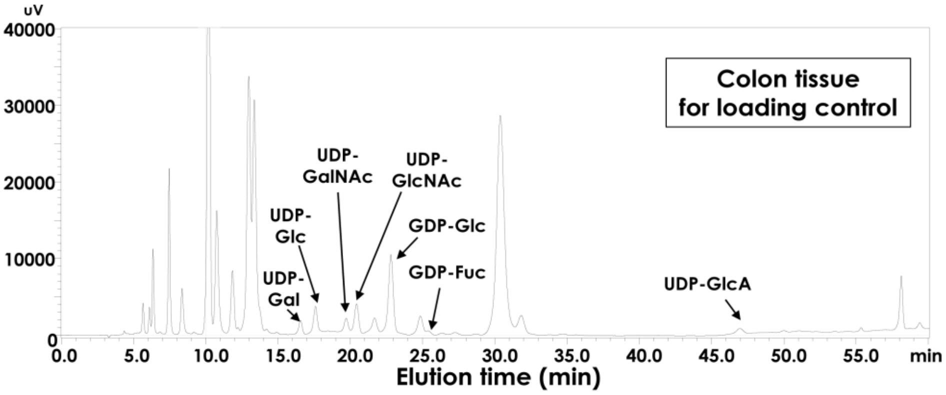

Supplement Fig. 1: Nucleotide sugars derived from colon tissue for loading control. Nucleotide sugars were separated by ion-pair reversed-phase HPLC. Each peak was identified by comparison with the retention times of a standard mixture.

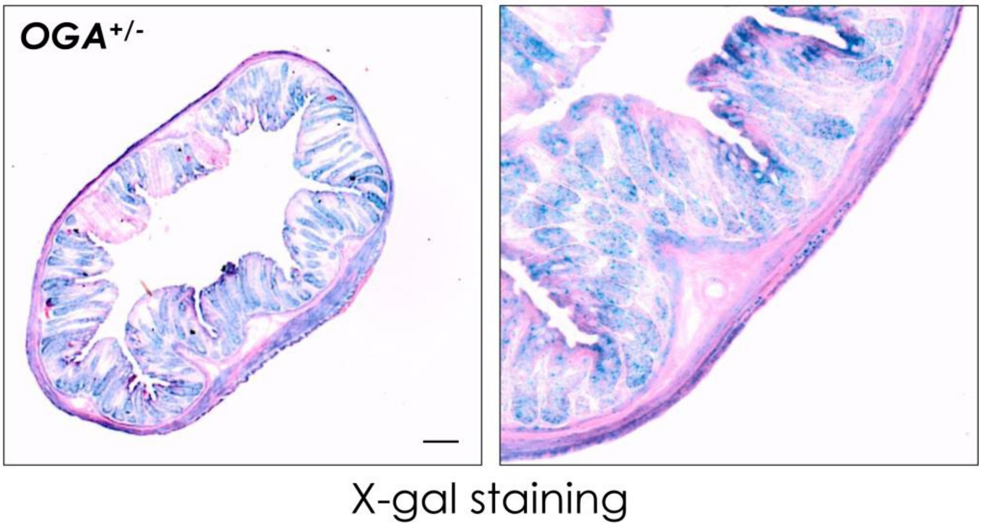

Supplemental Fig. 2: OGA expression in colon epithelial cells. Colon sections of *OGA*$^{+/-}$ mice were stained with X-gal. Bars =100 μm

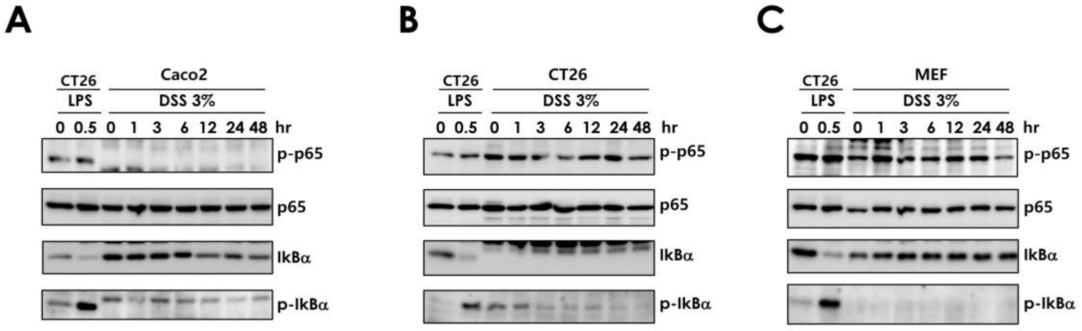

Supplement Fig. 3: Caco2, CT26 cells, and MEFs were treated with 3% DSS for up to 48 hours. NF-κB signaling were analyzed by Western blot analysis.

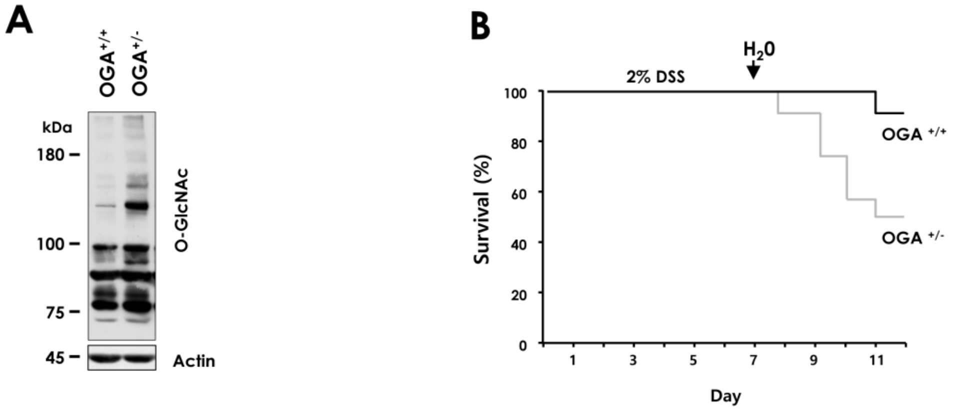

Supplemental Fig. 4: (A) Immunoblot analysis confirmed the O-GlcNAcylation levels in colon tissue lysates of the $OGA^{+/+}$ and $OGA^{+/–}$ mice. (B) Kaplan–Meier survival curve of $OGA^{+/+}$ and $OGA^{+/-}$ mice during DSS treatment and the recovery phase. n = 14 mice per group.

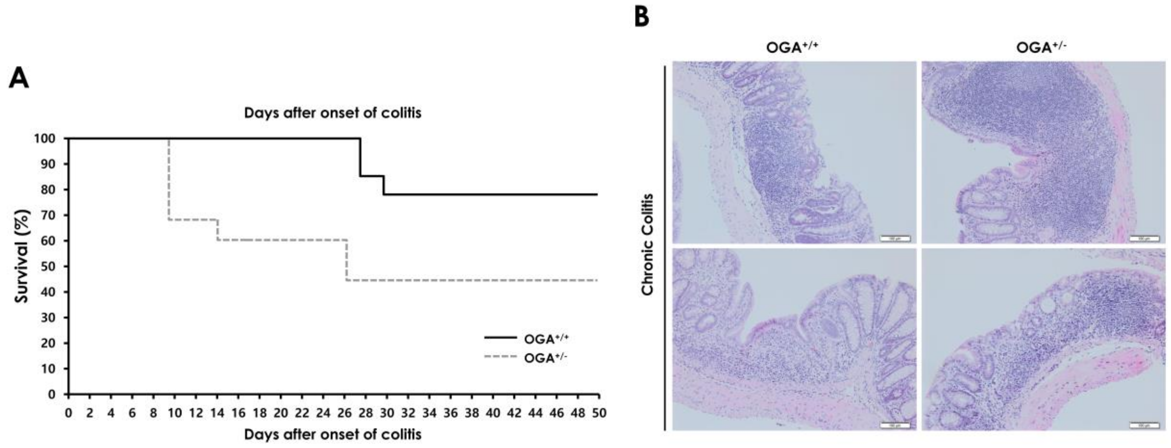

Supplemental Fig. 5: Chronic colitis of *OGA*$^{+/+}$ and *OGA*$^{+/-}$ mice (A) Kaplan–Meier survival curve of *OGA*$^{+/+}$ and *OGA*$^{+/-}$ mice during chronic colitis. (B) Representative pictures of colon sections. Bars=100 μm

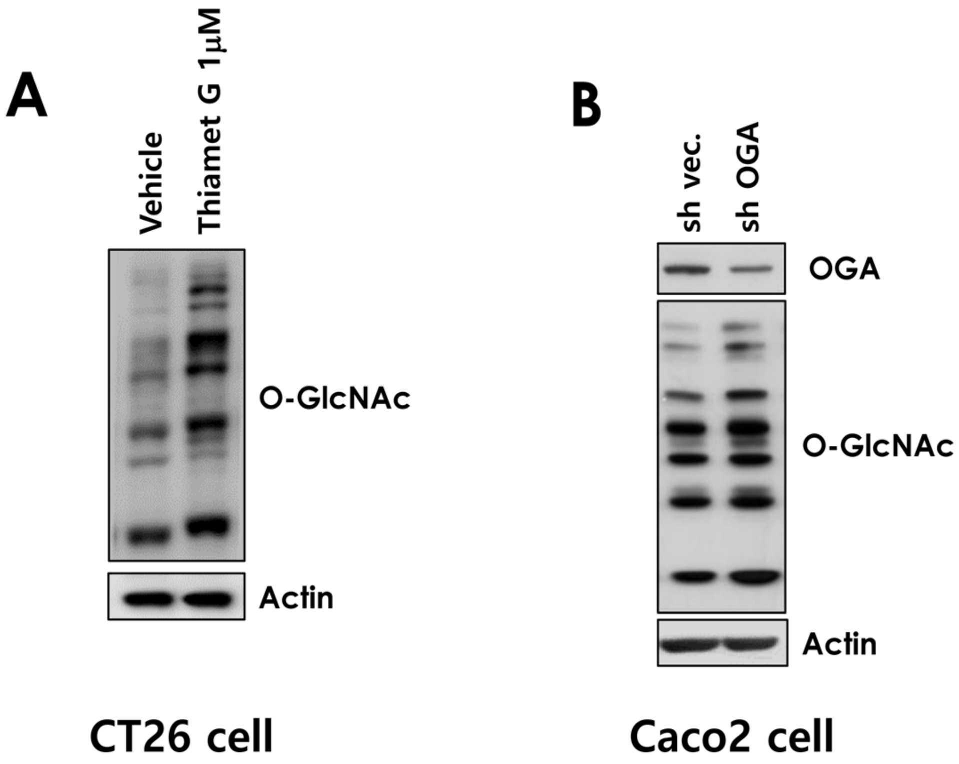

Supplemental Fig. 6: O-GlcNAc levels in OGA inhibitor treatment and OGA Knockdown cell lines (A) Thiamet G elevates O-GlcNAcylation in CT26 cells. CT26 cells were treated with Thiamet G for 18 hr. (B) Increased O-GlcNACylation in OGA Knockdown Caco2 cell lines. O-GlcNAc levels were analyzed by Western blot analysis with a RL2(O-GlcNAc) antibody.

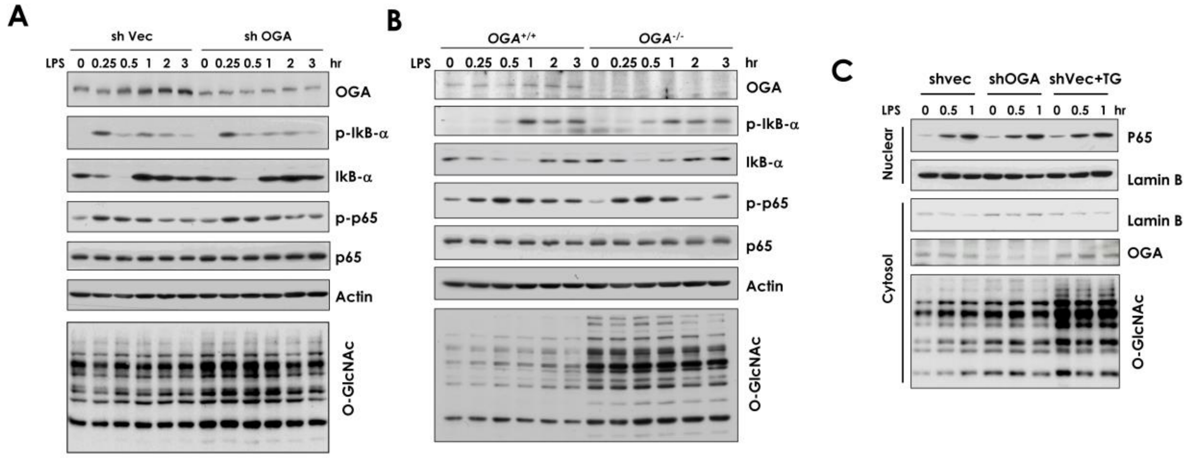

Supplemental Fig. 7: Increased O-GlcNAcylation doesn't affect Iκb degradation, p65 phosphorylation, and p65 nuclear translocation. (A) Control and OGA knockdown Caco2 cell lines were also incubated with LPS and NF-κB signaling was analyzed by Western blot analysis. (B) *OGA*$^{+/+}$ and *OGA*$^{-/-}$ MEFs were incubated with LPS and NF-κB signaling was analyzed by Western blot analysis. (C) Control, OGA knockdown, and Thiamet G-treated control cell lines were stimulated with LPS. The nuclear extracts were prepared and subjected to Western blot analysis.

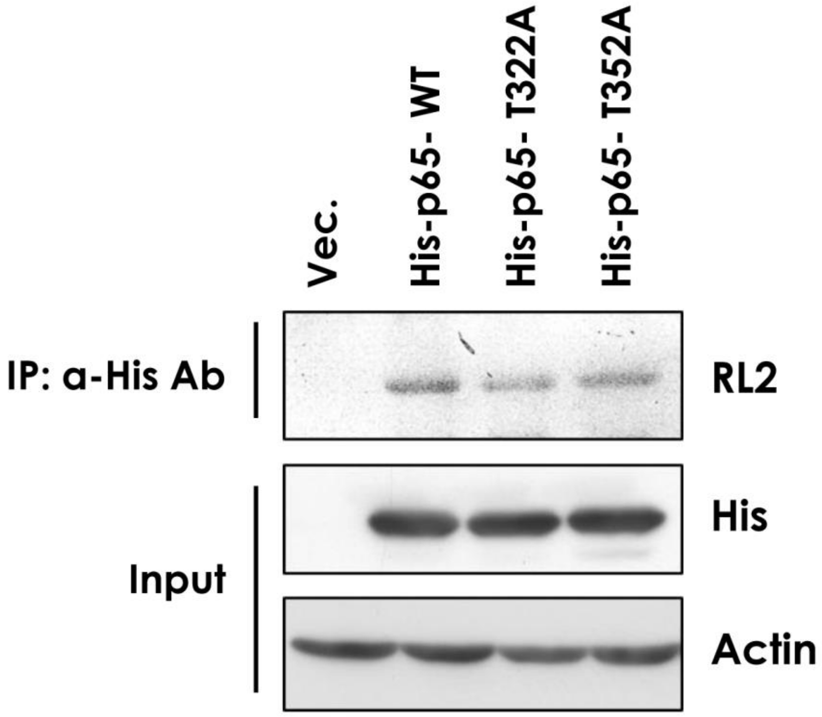

Supplemental Fig. 8: O-GlcNAcylation of p65 WT and p65 mutants. HEK-293 cells were transfected with the indicated His-p65 construct (WT, T322A, and T352A). O-GlcNAcylated p65 was detected by anti-O-GlcNAc antibody (RL2) after immunoprecipitation with anti-His antibody.
